# Triel Bonds, π-Hole-π-Electrons Interactions in Complexes of Boron and Aluminium Trihalides and Trihydrides with Acetylene and Ethylene

**DOI:** 10.3390/molecules200611297

**Published:** 2015-06-19

**Authors:** Sławomir J. Grabowski

**Affiliations:** 1 Kimika Fakultatea, Euskal Herriko Unibertsitatea UPV/EHU and Donostia International Physics Center (DIPC), P.K. 1072, 20080 Donostia, Spain; E-Mail: s.grabowski@ikerbasque.org; Tel.: +34-943-015-477; 2 Ikerbasque, Basque Foundation for Science, 48011 Bilbao, Spain

**Keywords:** boron and aluminium Lewis acid centres, π-hole, triel bond, Quantum Theory of “Atoms in Molecules”, Natural Bond Orbitals approach

## Abstract

MP2/aug-cc-pVTZ calculations were performed on complexes of aluminium and boron trihydrides and trihalides with acetylene and ethylene. These complexes are linked through triel bonds where the triel center (B or Al) is characterized by the Lewis acid properties through its π-hole region while π-electrons of C_2_H_2_ or C_2_H_4_ molecule play the role of the Lewis base. Some of these interactions possess characteristics of covalent bonds, *i.e.*, the Al-π-electrons links as well as the interaction in the BH_3_-C_2_H_2_ complex. The triel-π-electrons interactions are classified sometimes as the 3c-2e bonds. In the case of boron trihydrides, these interactions are often the preliminary stages of the hydroboration reaction. The Quantum Theory of “Atoms in Molecules” as well as the Natural Bond Orbitals approach are applied here to characterize the π-hole-π-electrons interactions.

## 1. Introduction

The hydrogen bond seems to be the most often analyzed Lewis acid-Lewis base interaction since its key role in numerous chemical, physical and biochemical processes is well known [1,2]. For example, it often may be considered as the preliminary stage of the proton transfer process [3] and it is often the main factor responsible for the arrangement of molecules in crystal structures [1,2,4]. However, there are other Lewis acid-Lewis base interactions which are important in numerous processes [5,6,7]; often they are important as initial stages of chemical reactions.

The σ-hole concept proposed in recent years to explain the nature of halogen bonds [8,9] is also applied to describe other interactions [10,11,12,13]. One can mention Groups 14–17 elements interacting as the Lewis acid centers with Lewis bases; such interactions are named as tetrel [14,15,16,17,18], pnicogen [19,20,21,22], chalcogen [23,24,25,26] and halogen bonds [27,28], respectively. The existence of those stabilizing interactions often seems to be surprising since numerous elements of the mentioned above groups are known as electronegative centers, thus possessing properties of Lewis bases. However, the σ-hole concept explains that they are characterized by the regions of depletion of the electron charge density on the extension of bonds to those centers (σ-holes) [10,11,12,13]. If the depletion is sufficient enough, these regions are characterized by the positive electrostatic potential (EP). Experimental and theoretical results confirm this concept since the directional links, *i.e.*, the σ-hole bonds, between these regions of the positive EP and the Lewis base centers are often observed. On the other hand, often those centers have the dual character since they act simultaneously as the Lewis acid and as the Lewis base. For example, the monovalent halogen atoms are characterized by the positive EP in the elongation of the bond to the halogen while perpendicularly to this bond, or nearly so, there is “the belt” of negative EP resulting from the lone electron pairs [8,9].

Another class of Lewis acid-Lewis base interactions, *i.e.*, π-hole bonds, was recently analyzed [11,13]. It was stated that π-hole is a region of low electron density which is situated in a direction perpendicular to a center of a planar molecule or a planar portion of a molecular framework [11]. The following atoms were mentioned as those characterized by the existence of π-holes; the boron in boron trihalides, the sulfur in SO_2_ molecule or the nitrogen in FNO_2_ [11]. Very recently, the directionality of π-holes in nitro compounds was analyzed [29]. Other examples concern the anion-π-hole interactions in crystal structures [30] or σ-holes and π-holes acting cooperatively in the Fmoc-Leu-ψ[CH_2_-NCS] crystal structure [31].

The triel centers (Group 13 elements) in trihydrides and trihalides were chosen here for analysis since they possess the strong Lewis acid properties in the direction perpendicular to the plane of the molecule due to the existence of the π-holes characterized by the positive EP [32,33]. The triel atom in the species mentioned above is an electron deficient center since it has six electrons in the outer shell—these are the electrons of three σ-bonds between the triel atom and hydrogen or halogen atoms. The electron deficient region is related to the outer vacant p orbital which is perpendicular to the plane of the molecule. In general, the deficiency of valence electrons is known as the hypovalency [34], and the octet rule is not obeyed here (less than eight electrons). The octet rule is not obeyed also for the hypervalent centers [34] where there is more than eight electrons in the valence shell.

**Figure 1:**
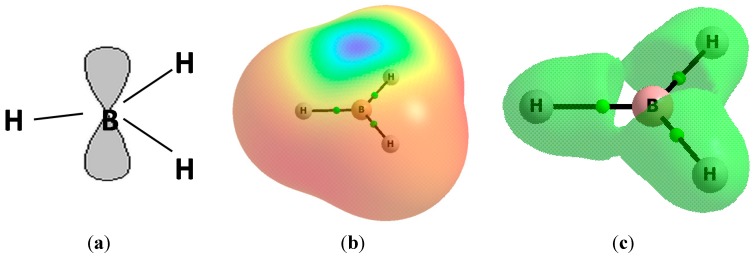
The BH_3_ molecule, (**a**) scheme showing the vacant p-orbital; (**b**) the electrostatic potential surface calculated for the 0.001 au electron density, red and blue colors correspond to negative and positive EP, respectively; (**c**) the molecular graph of the BH_3_ molecule with the reactive surface. Big circles correspond to atomic attractors and small green circles to the bond critical point; results of the MP2/aug-cc-pVTZ calculations.

Figure 1 presents the scheme of the BH_3_ molecule as an example where the vacant p-orbital perpendicular to the molecular plane is shown (Figure 1a); the figure also shows the electrostatic potential (EP) surface (Figure 1b) and the molecular graph with the reactive surface (Figure 1c), *i.e.*, with the isosurface where the laplacian of the electron density is equal to zero (∇^2^ρ = 0). One can see the region of the positive EP (π-hole) in the direction perpendicular to the plane of the molecule and situated above the B-atom position. The space closed by the reactive surface is characterized by the negative values of ∇^2^ρ, thus it corresponds to the concentration of the electron density. The region at the B-atom position is characterized by the depletion of the electron charge density (∇^2^ρ > 0, Figure 1c). Hence, one can see the positive EP as well as the positive ∇^2^ρ region which correspond to the vacant *p*-orbital and which show nucleophilic attack sites of the BH_3_ molecule. The EP and ∇^2^ρ similar distributions are observed for other boron and aluminum hydrides and halides—it means that the sites of the possible nucleophilic attack are observed at the B and Al positions.

The complexes of boron trihydrides and trihalides with Lewis bases were the subject of numerous investigations, and often strong interactions were found in those moieties [35,36]. The interactions of B, Al, Ga, In and Tl centers in trihalides and trihydrides with the nitrogen Lewis base centers were analyzed recently and it was described that often they possess characteristics of covalent bonds; those interactions were classified as the π-hole bonds [32]. Very often the triel-nitrogen link is as strong as the typical covalent bond and the triel center may be considered as the tetravalent one where the octet rule is obeyed [32,33].

The aim of this study is to analyze the triel-π-electrons interactions in complexes of boron and aluminium trihydrides and trihalides with acetylene and ethylene. The C_2_H_2_ and C_2_H_4_ π-electron systems were chosen here as Lewis bases since it was found in numerous studies that the π-electron systems often interact strongly with Lewis acids. For example, the role of π-electrons as the proton acceptor in A-H–π hydrogen bonds is very well known [4], and special attention was paid to C-H–π hydrogen bonds [37]. The π-electron systems may also interact with σ-holes; for example, the pnicogen–π interactions were analyzed in complexes of ECl_3_ (E = As, Sb, Bi) with aromatic species [38]; one can also mention the recent study on S–π chalcogen bonds [39,40]. The boron and aluminum species were chosen here since they are characterized by strong Lewis acidity; the complexes of triel trihydrides and trihalides with the conventional one center Lewis bases were analyzed before and it was found that the binding energies, E_bin_’s, for some complexes of aluminum and gallium are about 100 kJ/mol or even more (absolute values since the E_bin_ value for stabilizing interaction is negative) [32].

The complexes of the BH_3_ species with alkenes and alkynes are analyzed from time to time in terms of the hydroboration reactions. The hydroboration is defined as the addition of the H-B bond to double bonds, particularly to the C=C bond, as well as to the C≡C triple bond [41,42]. However, studies on the halogenoboration [43] and hydroalumination [44] have also been conducted. It means that the interactions analyzed here may be treated as the preliminary stages of the abovementioned processes similarly as the hydrogen bond initiates the proton transfer process [3] or the dihydrogen bond initiates the reaction of the release of the molecular hydrogen [45,46].

## 2. Results and Discussion

### 2.1. The Strength of π-Hole-π Electrons Triel Bonds

**Figure 2:**
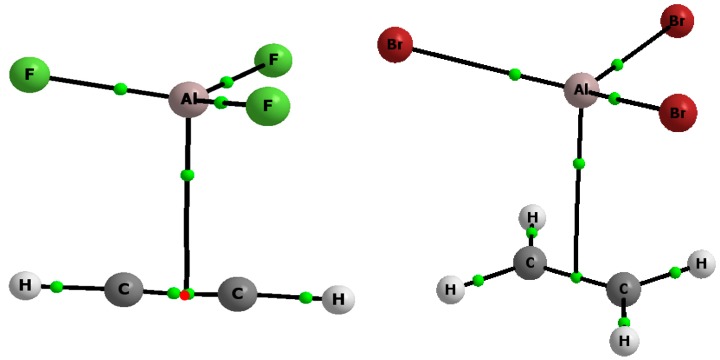

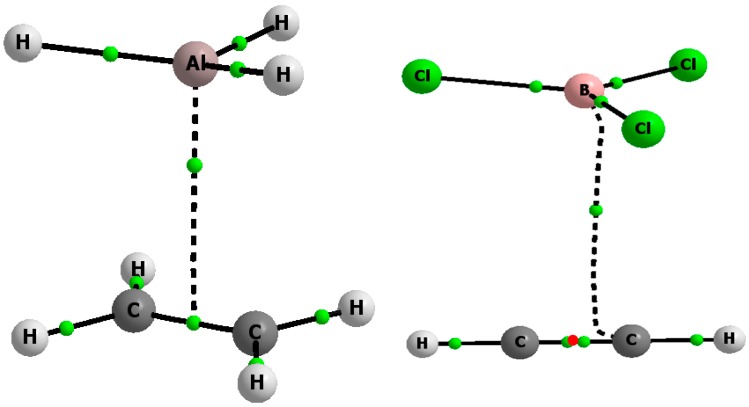
The molecular graphs of the AlF_3_-C_2_H_2_ (**the top left**), AlBr_3_-C_2_H_4_ (**the top right**), AlH_3_-C_2_H_4_ (**the bottom left**) and BCl_3_-C_2_H_2_ (**the bottom right**) complexes, big circles correspond to attractors, small green circles to the bond critical points, small red circles to the nonnuclear attractors and the solid and broken lines to the bond paths.

Figure 2 shows the bond paths between the Al or B center and the π-electrons of CC bond of acetylene or ethylene. This may suggest that mainly the π-hole-π-electrons contacts are responsible for the stabilization of the complexes formed. The BH_3_-C_2_H_4_ complex is not presented since optimizations with differently started configurations of this system led to the CH_3_-CH_2_-BH_2_ molecule. It may mean that the hydroboration reaction takes place here and that the stable BH_3_-C_2_H_4_ complex linked by the triel bond does not exist. The similar situation was observed here for the BH_3_-B_2_H_4_ complex initially constructed to be linked through B…π(BB) contact; however, the optimization led to the stable B_3_H_7_ species corresponding to the energetic minimum. Early calculations on the hydroboration reaction have shown the BH_3_-C_2_H_4_ complex is stable [47,48] with the binding energy of about −10 kJ/mol. However, the HF/6-31G(d,p) level was applied for those calculations [48]. The more systematic MP2/6-311G(2df,2pd)//MP2/TZ2P calculations were performed for the BH_3_ and BX_3_ (X = F, Cl) complexes with the H_2_, C_2_H_2_ and C_2_H_4_ Lewis bases [49]. These calculations show the BCl_3_ < BF_3_ < BH_3_ Lewis acidity trend for the boron center while the Lewis basicity shows the trend H_2_ < C_2_H_2_ < C_2_H_4_ [49,50]. It is interesting that the MP2/6-31G(d,p) calculations performed on the BH_3_-C_2_H_4_ complex [49] have shown that it is stable with the binding energy of about −40 kJ/mol; it is a significant difference in comparison with the previous Hartree-Fock calculations [48]. However, the barrier for the rearrangement yielding ethylborane CH_3_-CH_2_-BH_2_ is negligible here since it is less than 0.1 kcal/mol [49]. Hence, it is not surprising that for the higher level calculations performed here, the minimum corresponding to the stable BH_3_-C_2_H_4_ complex is not observed.

The infrared spectra of BF_3_/C_2_H_4_ and BF_3_/C_3_H_6_ mixtures dissolved in liquid argon and liquid nitrogen were analyzed and it was found that the BF_3_ molecule binds to the C=C double bond forming van der Waals complexes [51]. It is worth mentioning that the detailed NBO analysis on the structure of the BH_3_-C_2_H_4_ complex was performed and it was found that the interaction between the boron center and π-electrons of ethylene is very strong and may be treated as the three center—two electron (3c-2e) covalent bond [34] which may be represented by the three center bond orbital [52]. One can see that the triel center-π-electrons interactions in ZH(X)_3_-π complexes are analyzed from time to time. However, they mainly concern the boron center (Z = B) and the light halogen atoms (X = F, Cl) and corresponding early analyses were performed on low levels calculations. This is why the high level MP2/aug-cc-pVTZ calculations were carried out here and the sample contains not only boron complexes but also aluminum ones; additionally, the bromine is included in the ZX_3_ series of the Lewis acid units.

Table 1 presents the interaction and binding energies for the complexes analyzed here. The BSSE corrected energies are also included as well as the deformation energies (energies are defined in the section 3). The stronger interactions are observed for aluminum complexes than for boron ones; the −E_int_BSSE value for aluminum complexes is from 47.7 kJ/mol to 83.9 kJ/mol while for boron complexes from 11.0 kJ/mol to 14.0 kJ/mol, except of the BH_3_-C_2_H_2_ complex which is characterized by the strong interaction since −E_int_BSSE amounts to 69.9 kJ/mol. The distances between the triel center and the carbon atom of ethylene or acetylene are presented. For each complex, two triel-carbon distances are almost equal to each other; thus the mean Z…C distances are shown. These distances roughly reflect the strength of interactions since for shorter distances stronger interactions are observed. For the aluminum species, the Z…C distance amounts to ~2.5 Å, and for the boron complexes it is equal to ~3.0–3.3 Å. The BH_3_-C_2_H_2_ complex is an exception again since this distance is equal to ~2 Å.

**Table 1:** The energetic parameters (in kJ/mol), the mean triel-carbon distance, R (in Å), and the ∑α parameter (in degrees), E_int_BSSE and E_bin_BSSE are E_int_ and E_bin_ energies corrected for BSSE; the maximum electrostatic potential (EP) for ZH_3_ and ZX_3_ monomers is included.

Complex	EP	E_int_	E_int_BSSE	E_bin_	E_bin_BSSE	E_def_	∑α	R
AlBr_3_-C_2_H_2_		−81.8	−68.9	−60.6	−47.4	21.2	351.4	2.482
AlBr_3_-C_2_H_4_	0.103	−94.3	−77.9	−71.1	−54.7	23.2	350.8	2.497
AlCl_3_-C_2_H_2_		−79.6	−73.2	−58.2	−51.9	21.3	351.9	2.479
AlCl_3_-C_2_H_4_	0.122	−89.5	−81.6	−66.3	−58.4	23.2	351.1	2.502
AlF_3_-C_2_H_2_		−84.7	−78.3	−66.9	−60.5	17.8	353.8	2.437
AlF_3_-C_2_H_4_	0.202	−91.6	−83.9	−72.6	−64.9	19.0	353.5	2.467
AlH_3_-C_2_H_2_		−50.2	−47.7	−43.3	−40.7	7.0	357.0	2.562
AlH_3_-C_2_H_4_	0.130	−58.1	−55.1	−50.5	−47.4	7.7	356.7	2.552
BBr_3_-C_2_H_2_		−18.6	−11.0	−18.4	−10.8	0.2	359.9	3.325
BBr_3_-C_2_H_4_	0.034	−22.8	−12.2	−22.5	−11.9	0.3	359.9	3.288
BCl_3_-C_2_H_2_		−14.2	−11.3	−14.0	−11.0	0.3	359.9	3.312
BCl_3_-C_2_H_4_	0.043	−16.5	−12.6	−16.1	−12.3	0.3	359.9	3.297
BF_3_-C_2_H_2_		−16.4	−12.9	−15.4	−12.0	0.9	359.9	2.994
BF_3_-C_2_H_4_	0.090	−18.2	−14.0	−17.0	−12.7	1.3	359.7	2.990
BH_3_-C_2_H_2_	0.070	−74.2	−69.2	−36.2	−31.2	38.0	348.4	2.014

Table 1 presents the maximum EP values for the 0.001 au molecular surfaces of the ZH_3_ and ZX_3_ molecules not involved in interactions. Those maxima occur at the Z-centers (Figure 1b). The EP maximum values are systematically greater for aluminum species than for the boron ones. The EP values for Al and B monomers show the trend Br < Cl < H < F. This trend is in agreement with the increase of the electronegativity of substituents if only halogens are taken into account; it may mean that for more electronegative halogens, the greater electron density shift from the Z center to the substituent should be observed. However, the triel trihydrides do not follow the aforementioned trend. Similarly, for the same Lewis base (C_2_H_2_ or C_2_H_4_), the strength of interaction in complexes increases for more electronegative substituent, the H < Br < Cl < F trend is observed for Al complexes while for the boron complexes, the BH_3_-C_2_H_2_ complex does not follow the trend. The observations on the strength of interaction are based on the −E_int_BSSE and −E_bin_BSSE values (Table 1).

**Figure 3:**
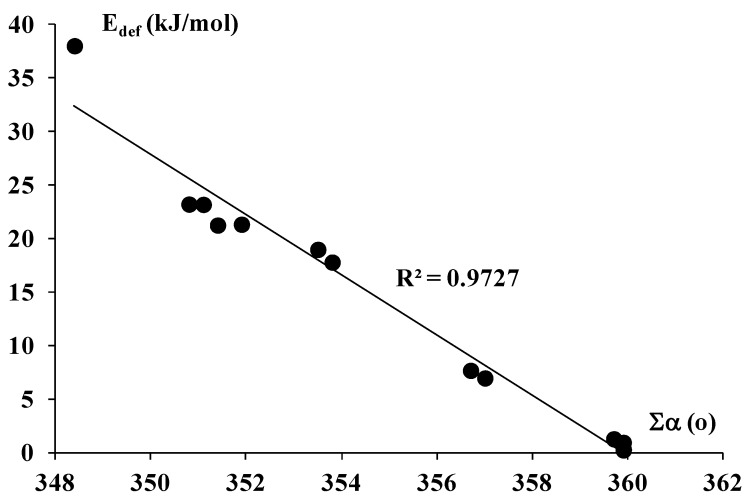
The linear correlation between the ∑α parameter (in degrees) and the deformation energy (in kJ/mol).

The aforementioned results may explain why the Al complexes are linked by stronger interactions than the B ones and why the Br < Cl < F trend is observed for the strength of interaction in Al and B series. This may also suggest that the strength of interaction depends mainly on EP value at Z center and thus it is ruled by the electrostatic interactions. However, one can see that the deformation energy for the AlX_3_ complexes amounts to ~20 kJ/mol, less for AlH_3_ complexes, ~7–8 kJ/mol, for the BH_3_-C_2_H_2_ complex it is equal to 38 kJ/mol while for the other boron complexes this value amounts to ~1 kJ/mol or even less. The deformation energy is related to geometrical changes connected with the transformation from the trivalent triel structure in isolated ZX_3_ and ZH_3_ species to the triel structure in complexes. For the ideal trigonal ZX_3_ or ZH_3_ structure, the sum of three X-Z-X (H-Z-H) angles is equal to 360° while for the ideal tetravalent structure characterized by the sp^3^ hybridization where the fourth connection (interaction) with Z-center possesses properties of the covalent bond, this sum should correspond to the hybridization; *i.e.*, it should be equal to ~327°. Hence, one may expect that the stronger, more covalent interaction of the Lewis base with the ZX_3_ or ZH_3_ species results in lower above-mentioned sum of angles (designated later here as ∑α). Table 1 shows the lowest ∑α value for the BH_3_-C_2_H_2_ complex and again the ∑α values are systematically greater (close to 360°) for the remaining boron complexes than for the aluminum ones where this value is situated in the 350°–357° range. Figure 3 presents good correlation between the deformation energy and the ∑α value.

The deformation energy reflecting geometrical changes being the result of complexation is connected with the electron charge redistribution (electron charge density shift). Hence, it means that the differences between the Al complexes and the BH_3_-C_2_H_2_ complex on one hand and the remaining boron complexes on the other hand result mainly from the electron charge shifts related to charge transfer and polarization interactions and that these differences do not result from the electrostatic interactions as the EP values (Table 1) could suggest. The above-mentioned charge transfer and polarization interactions are usually attributed to covalence or to the partly covalent character of inter- and intramolecular links [3,34]. It is also interesting why for the Al complexes stronger interactions are observed than for the boron ones; and why distinct characteristics are detected for the BH_3_-C_2_H_2_ complex if it is compared with the other boron complexes. It is discussed in detail later here.

Table 1 shows that the interactions with ethylene are systematically stronger than their counterparts with acetylene; this is in line with the gas basicity values amounting to 616.7 and 651.5 kJ/mol for acetylene and ethylene, respectively [53]. Table 1 shows the E_def_ values are systematically greater for complexes with ethylene than for their counterparts with acetylene; it means that the ethylene as the stronger Lewis base causes greater structural changes in complexes than the acetylene. For example, for the AlF_3_-C_2_H_4_ complex, the deformation energy is equal to 19.0 kJ/mol while for the AlF_3_-C_2_H_2_ it amounts 17.8 kJ/mol. Even for boron, the small difference in E_def_ is observed between ethylene and acetylene complexes. Only for both complexes of BCl_3_ is the deformation energy practically the same since it is equal to 0.3 kJ/mol.

### 2.2. The Analysis of QTAIM Parameters

Table 2 presents selected characteristics of the bond critical point (BCP) of the bond path linking the Z-triel center with the π-electron system of acetylene or ethylene. For some complexes, there is the bond path (BP) between the Z-attractor and the BCP of CC bond (Figure 2). For other systems, there is the BP between the Z-attractor and the non-nuclear attractor (NNA) situated between two BCPs of the CC bond (Figure 2). It is worth mentioning that NNA and two neighboring BCPs of the CC bond are very close to each other and they are characterized by similar values of the electron density. It seems that the existence of the NNA and two surrounding BCPs may be characteristic for some of π-electron systems and that sometimes, because of the proximity of those points, only one is observed, *i.e.*, BCP. For the BCl_3_-C_2_H_2_ complex, the bond path between the B-attractor and the C-attractor of acetylene is observed (Figure 2). It was found earlier that for some A-H…π hydrogen bonds, the bond paths between the H-attractor of the proton donating A-H bond and the BCP of the π-electron system exist while for other A-H–π interactions the H-attractor–C-attractor bond paths link the Lewis acid and Lewis base units [54].

**Table 2:** The characteristics of the bond critical point (in au) corresponding to the bond path linking the Z-center with the π-electron system; the electron density at BCP, ρ_BCP_, its laplacian, ∇^2^ρ_BCP_, and the total electron energy density at BCP, H_BCP_.

Complex	ρ_BCP_	∇^2^ρ_BCP_	H_BCP_
AlBr_3_-C_2_H_2_	0.0276	0.0818	−0.0035
AlBr_3_-C_2_H_4_	0.0292	0.0725	−0.0049
AlCl_3_-C_2_H_2_	0.0272	0.0847	−0.0031
AlCl_3_-C_2_H_4_	0.0284	0.0735	−0.0044
AlF_3_-C_2_H_2_	0.0275	0.1036	−0.0017
AlF_3_-C_2_H_4_	0.0283	0.0903	−0.0030
AlH_3_-C_2_H_2_	0.0212	0.0683	−0.0014
AlH_3_-C_2_H_4_	0.0234	0.0687	−0.0023
BBr_3_-C_2_H_2_	0.0068	0.0191	0.0008
BBr_3_-C_2_H_4_	0.0073	0.0195	0.0007
BCl_3_-C_2_H_2_	0.0064	0.0183	0.0007
BCl_3_-C_2_H_4_	0.0070	0.0182	0.0007
BF_3_-C_2_H_2_	0.0092	0.0273	0.0010
BF_3_-C_2_H_4_	0.0101	0.0264	0.0008
BH_3_-C_2_H_2_	0.0620	0.0325	−0.0392

**Figure 4:**
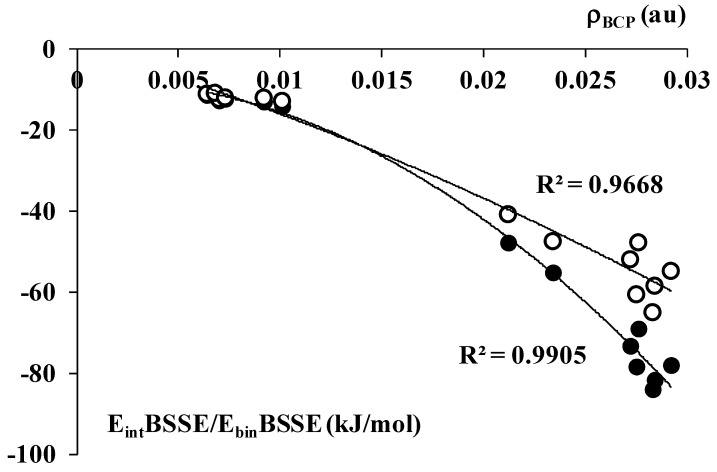
The second order polynomial relationships between the electron density at BCP, ρ_BCP_ (in au), and the E_int_BSSE as well as E_bin_BSSE, black and white points, respectively (energies in kJ/mol). The E_int_BSSE and E_bin_BSSE are corrected for BSSE and they correspond to E_int_ and E_bin_ defined by Equations (1) and (2), respectively.

Table 2 shows greater ρ_BCP_ values for aluminum complexes, ~0.02–0.03 au, than for boron ones, ~0.01 au. The BH_3_-C_2_H_2_ complex is an exception similarly as for the other parameters described in the previous section since it is characterized by the ρ_BCP_ value of 0.06 au. Figure 4 shows the correlations between the ρ_BCP_ value and the interaction/binding energy. The BH_3_-C_2_H_2_ complex is excluded from those correlations which is statistically justified since it is characterized by the ρ_BCP_ value being outside of the range of the remaining species. Such approach of the exclusion of single species from the regression analysis is statistically justified if it concerns the values very distant from the other values of the whole sample considered.

It was shown in early studies, especially for H-bonded systems, that the ρ_BCP_ expresses the strength of interaction for samples of related species [3]. This is also in force for the systems analyzed here since the second order polynomial dependence between the electron density at BCP and the interaction/binding energy is observed (Figure 4). The difference between the binding and interaction energies (deformation energy) increases if the ρ_BCP_ value increases. This means that the greater geometrical changes in the system and, in consequence, the greater deformation energy is observed with the increase of the strength of interaction. Such dependencies were also observed earlier for the hydrogen bonded systems [55].

Table 2 shows the negative values of the total electron energy density at BCP, H_BCP_, for the BH_3_-C_2_H_2_ complex and for aluminium complexes. For the remaining boron complexes, this value is positive. It was pointed out in early studies that the negative H_BCP_ value indicates the covalent character of interaction between atoms connected by the corresponding bond path; at least such an interaction is characterized by the partial covalence [56,57]. It was also found for the hydrogen bond systems that the contribution of the covalent character increases with the strength of interaction [3]. This is also observed for the complexes linked through the triel bonds analyzed here since the strongest interactions are observed for aluminum complexes and for the BH_3_-C_2_H_2_ complex (Table 1, see E_int_ and E_bin_ energies). One can see that the negative values of the total electron energy density at the bond critical point, H_BCP_, which show the partial covalence of interactions, correspond to those complexes where the greatest E_def_’s are observed. The latter energies are related to the geometrical changes approaching the sp^3^ hybridization of the triel center.

### 2.3. The NBO Analysis and the Electron Charge Redistributions being the Result of Complexation

Table 3 presents NBO atomic charges of the ZX_3_ and ZH_3_ species not involved in interactions (Z_mon_, X_mon_, H_mon_) and the corresponding atomic charges for these species in the complexes (Z_com_, X_com_, H_com_). The X (or H) atomic charges of each ZX_3_ (or ZH_3_) moiety considered are almost equal to each other and thus the mean values are shown in the table (averaging over three X/H substituent atoms). The ET electron charge transfer values from the Lewis base (acetylene or ethylene) to the Lewis acid (triel trihydride or trihalide) are also shown. The ET values also represent the charges of the ZX_3_ and ZH_3_ triel species in complexes. The E_NBO_^1^ and E_NBO_^2^ energies are presented; they correspond to the π_CC_ → n_Z_* and π_CC_ → n_ZX(H)_* orbital-orbital interactions, respectively. These interactions represent the most important orbital–orbital overlaps for the complexes analyzed here.

The greatest electron charge transfer, ET, is observed for the BH_3_-C_2_H_2_ complex. The ET-values are greater for Al-complexes than for the remaining boron species. One can see that the ET values are in line with the other parameters analyzed earlier here, interaction and binding energies and the BCP characteristics. It means that roughly the greater ET values correspond to stronger interactions.

The complexation leads to the decrease of the positive charge of the Z-center for the aluminum complexes and for the BH_3_-C_2_H_2_ complex. In the latter case, there is a huge decrease since the positive charge of boron in the isolated BH_3_ species changes into the negative one in the complex. For the remaining boron complexes, the increase of the positive charge of boron is observed as a result of complexation. One can see here the difference between complexes with stronger interactions (H_BCP_ negative) and those weakly bonded (positive H_BCP_). The NBO results for hydrogen and halogen substituents are ambiguous. The H-charges are much less negative after complexation in the BH_3_-C_2_H_2_ complex. Thus, the complex formation is connected in this case with the outflow of the electron density from the Lewis base as well as from the hydrogen atoms of BH_3_ to the boron center. The similar decrease of the negative charge of H-atoms is observed in complexes of AH_3_; in complexes of AF_3_, the negative charge of F-substituents practically does not change after complex formation. However, for the remaining aluminum and boron complexes, the triel bond formation leads to the increase of the negative charge of substituents.

**Table 3:** The NBO charges of Z and X (or H) atoms in monomers and in complexes, ET—Electron charge transfer (values in au), E_NBO_^1^ and E_NBO_^2^ energies (in kJ/mol) correspond to the π_CC_ → n_Z_* and π_CC_ → n_ZX(H)_* orbital–orbital interactions.

Complex	E_NBO_^1^	E_NBO_^2^	Z_com_	Z_mon_	X/H_com_	X/H_mon_	ET
AlBr_3_-C_2_H_2_	314.6	77.8	1.230	1.333	−0.458	−0.444	−0.145
AlBr_3_-C_2_H_4_	346.7	79.7	1.210		−0.460		−0.169
AlCl_3_-C_2_H_2_	305.4	73.4	1.479	1.591	−0.540	−0.530	−0.142
AlCl_3_-C_2_H_4_	334.2	75.2	1.461		−0.542		−0.164
AlF_3_-C_2_H_2_	296.4	0	2.273	2.381	−0.791	−0.794	−0.101
AlF_3_-C_2_H_4_	335.6	0	2.258		−0.792		−0.117
AlH_3_-C_2_H_2_	221.5	34.0	1.161	1.315	−0.422	−0.438	−0.104
AlH_3_-C_2_H_4_	259.0	40.7	1.121		−0.415		−0.124
BBr_3_-C_2_H_2_	0	17.7	0.153	0.127	−0.053	−0.043	−0.008
BBr_3_-C_2_H_4_	0	22.9	0.151		−0.054		−0.011
BCl_3_-C_2_H_2_	13.8	0	0.465	0.442	−0.177	−0.147	−0.006
BCl_3_-C_2_H_4_	21.5	0	0.464		−0.158		−0.010
BF_3_-C_2_H_2_	17.9	1.6	1.582	1.569	−0.530	−0.523	−0.007
BF_3_-C_2_H_4_	25.7	2.9	1.580		−0.530		−0.012
BH_3_-C_2_H_2_	997.9	34.7	−0.066	0.418	−0.069	−0.139	−0.273

In the case of the ZX(H)_3_ monomers and the corresponding complexes, the H < Br < Cl < F trend of the increase of the positive charge of Al center is observed. It is in agreement with the increase of the electronegativity of the X/H substituent. The same trend is observed for boron trihalides and related complexes; however, the BH_3_ species and its acetylene complex are outside of the trend of the increasing electronegativity. One can see that aluminum centers are systematically more positive than their boron counterparts; it may be explained by a lower electronegativity of aluminum than of boron (1.61 and 2.04, respectively in the Pauling scale [58]).

**Figure 5:**
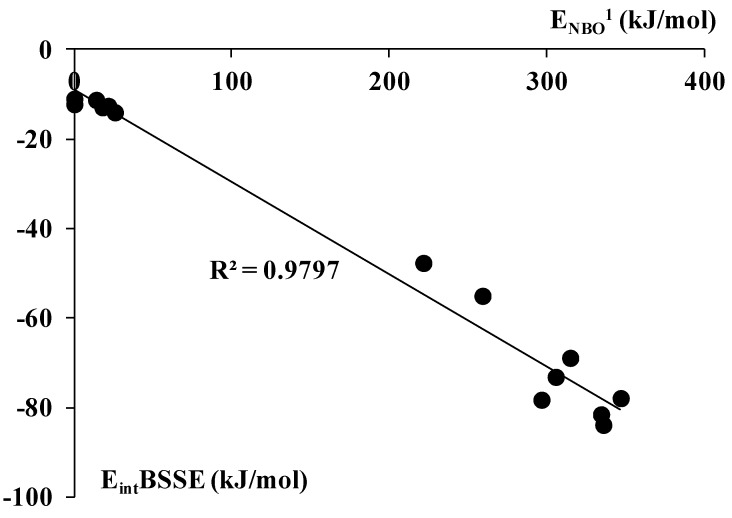
The correlation between the π_CC_ → n_Z_* orbital–orbital interaction energy (designated as E_NBO_^1^) and the E_int_BSSE (this is E_int_ defined in section 3 and next corrected for BSSE); both values in kJ/mol.

The π_CC_ → n_Z_* orbital–orbital interaction mentioned earlier here is one of the most important contributions to the charge transfer interaction; it is connected with the vacancy of p-orbital being perpendicular to the plane of BX(H)_3_ molecule. Figure 5 confirms the importance of the π_CC_ → n_Z_* overlap since good correlation between the total interaction energy and this orbital–orbital interaction energy is observed. The unusual properties of the BH_3_-C_2_H_2_ complex are also observed for the π_CC_ → n_Z_* orbital–orbital interaction energy which amounts to ~10^3^ kJ/mol (this complex is not included in the correlation presented in Figure 5 for the reasons explained for Figure 4). For the remaining boron complexes, this energy amounts to only ~10–20 kJ/mol and this interaction is not detected for BBr_3_ complexes. For the Al complexes, the energy is situated in the ~200–300 kJ/mol range. The properties of the BH_3_ molecule as a strong electron acceptor are in line with recent studies on the triel bonds [33] as well as with the study on the BH_3_-H_2_ complex [59] where the side-on coordinated conformation was found theoretically to be stable.

Table 3 presents also the E_NBO_^2^ energies corresponding to the π_CC_ → n_ZX(H)_* overlaps. These interactions are also important for the complexes analyzed here; however, they are not like the π_CC_ → n_Z_* interactions. In the BBr_3_ complexes, the latter interactions are not observed but the previous ones are detected. It may result from the large bromine substituents spheres which cover the small boron atom (and its vacant p-orbital) and thus the interaction with the antibonding n_B_* orbital is hindered here. However, the interaction with the n_BBr_* orbital is observed. On the other hand, the π_CC_ → n_AlF_* overlaps are not observed for the AlF_3_ complexes. This is due to strong polarizations of the Al-F bonds what result in the most positive aluminum charge (+2.26–2.27 au) in the AlF_3_ complexes in comparison with the other aluminum moieties; note that in the NBO approach the Al-F σ-bonds are not formed but there is the interaction between Al and F ions.

It was mentioned earlier here that the BH_3_-C_2_H_4_ complex optimized at lower levels of calculations was analyzed and it was found that the 3c-2e (three-center two-electron) bond orbital exists between boron and two carbon atoms of ethylene [34]; this orbital was designated as τ_CBC_. The 3c-2e bond orbital was also found for the BH_3_-H_2_ complex (τ_HBH_) and for other non-boron species [34]. The 3c-2e bond orbitals were found for the aluminum complexes as well as for the BH_3_-C_2_H_2_ complex analyzed here, *i.e.*, for those complexes where the strongest interactions are observed. The boron contribution in the τ_CBC_ bond is 16.2% in the BH_3_-C_2_H_2_ complex and the aluminum contribution in the τ_CAlC_ bond amounts to 4.8%–8.2% in the aluminum complexes. The contribution in the 3c-2e bond means the percentage of the electron density of the bond orbital localized at the center is considered. Thus, the above results show that the 3c-2e bonds are strongly polarized with the concentration of electron density at the Lewis base units (acetylene and ethylene). However, these results also show that the triel bonds in the BH_3_-C_2_H_2_ and aluminum complexes possess the covalent character, similarly as it was found within the QTAIM approach (negative H_BCP_ values—Table 2). It is worth mentioning that the restriction of the NBO approach to the description of the systems analyzed by two center orbitals results for these complexes in strong orbital–orbital interactions characterized by large energies (Table 3—E_NBO_^1^ values).

The results presented here indicate that for the aluminum complexes as well as for the BH_3_-C_2_H_2_ complex interactions related to the electron charge density shifts are the most important attractive ones. One can observe here large ET electron charge transfer values, large values of the orbital–orbital interaction energies (or the existence of 3c-2e bonds) and significant geometry deformations expressed by the deformation energies and the sum of X-Z-X (or H-Z-H) angles (∑α). The aforementioned parameters for the remaining boron complexes indicate weaker interactions characterized by negligible electron charge redistribution being the result of complexation.

What is the reason for much stronger interactions for the aluminum complexes and the BH_3_-C_2_H_2_ complex than for the remaining boron complexes? It was pointed out earlier here that the greater positive EP values and the greater atomic charges for Al than for B may be explained by the lower electronegativity of aluminum than of boron. However, it may explain only the stronger electrostatic interactions for the aluminum complexes than for the boron ones, and the electrostatic interaction is not an important attractive contribution to the total interaction, especially for the aluminum species. Since the interactions related to electron charge density shifts are more important here, the question should be why such shifts are more significant for aluminum species.

**Figure 6:**
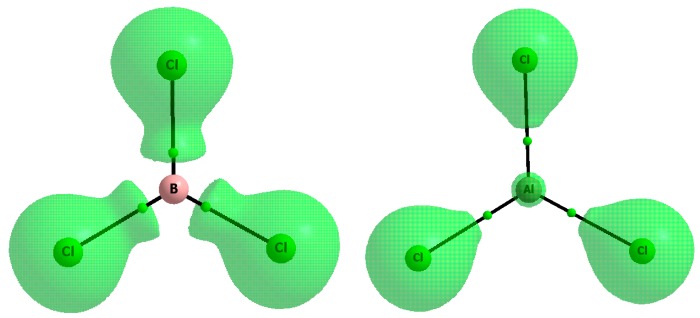
The molecular graphs of the BCl_3_ (left) and AlCl_3_ (right) molecules; solid lines correspond to bond paths, big circles to attractors and small green circles to BCPs, the reactive surfaces (∇^2^ρ(r) = 0 isosurfaces) for these molecules are presented; results of the MP2/aug-cc-pVTZ calculations.

The difference between boron and aluminum species is related to the electron structures of triel trihydrides and triel trihalides. Figure 6 shows the reactive surfaces (∇^2^ρ(r) = 0 isosurfaces) for BCl_3_ and AlCl_3_. The areas enclosed by such surfaces are characterized by the negative laplacian values; thus they correspond to the regions of the electron density concentration. For the remaining molecular space, the positive laplacian values are observed (∇^2^ρ(r) > 0). For the BCl_3_ molecule, three boron valence electrons are involved in the B-Cl σ-bonds. This is why the region of the positive ∇^2^ρ(r) is observed for the boron center and it corresponds to the location of the vacant p-orbital as well as to the location of the π-hole characterized by the positive EP. However, this small electrophilic region is compressed between large nucleophilic Cl-substituents regions which hinders interactions with Lewis bases. In the case of the AlCl_3_ molecule, the small region of the negative ∇^2^ρ(r) at the Al center is observed (Figure 6) due to the core electrons. However, the vacant p-orbital is characteristic for this structure, similarly as for other triel trihalides and trihydrides; the positive EP region at Al center is also observed. Consequently, the large area at the Al center is preferred for nucleophilic attack. That is why the Lewis bases usually are closer to the Al center than being covered by substituents B center. The latter proximity results in the greater electron density shifts for Al complexes than for the B ones.

The distinct situation is observed for the BH_3_ molecule where small H-substituents allow the Lewis bases to get closer to the boron center; it results in the enormous electron density shift to this center. It seems that the unusual properties of the BH_3_ species were not explained definitively. For example, the back bonding effect [60] was proposed to explain the weaker Lewis acid properties of boron trihalides than of boron trihydride. This effect exists for some of triel trihalides but it does not exist for the BH_3_ molecule. It is connected with the electron charge density shift from the halogen substituents to the triel center which results in the weakening of the Lewis acid properties of this center. However, the significance of back bonding was contested even in early studies [50]; recent studies on triel trihalides and trihydrides also indicate that the back bonding effect does not play the key role in the Lewis acid properties of the triel center [32,33]. Also, the results presented here are questioning its relevance, see for example the B-atomic charge and the EP at this center for BF_3_ and BH_3_ species; they are greater for the BF_3_ molecule (Table 1 and Table 3).

It seems that one of the possible factors influencing the strength of interactions between BH_3_ species and Lewis bases is connected with the low Lewis base properties of H-atoms [33]; the earlier study show the low negative EP value for H-atoms in boron trihydride. It results in the negligible repulsive forces between H-atoms of BH_3_ and Lewis base center, and consequently in the close boron-Lewis base contact and the large orbital–orbital overlap (or 3c-2e bond orbital formation); note that for the BH_3_-C_2_H_2_ complex the π_CC_ → n_Z_* interaction energy is extremely large, ~1000 kJ/mol (Table 1).

## 3. Experimental Section

MP2/aug-cc-pVTZ calculations were performed with the Gaussian09 set of codes [61] on the complexes of triel trihydrides, ZH_3_, and triel trihalides, ZX_3_, with acetylene and ethylene, where the Z triel atom is boron or aluminum while the X halogen atom is fluorine, chlorine or bromine. Figure 2 presents molecular graphs of the selected complexes analyzed here. The geometry optimizations performed on those complexes as well as on the monomers (ZH_3_, ZX_3_, C_2_H_2_ and C_2_H_4_) led to energetic minima since no imaginary frequencies were observed for them. For the ZBr_3_…C_2_H_2_ and ZBr_3_…C_2_H_4_ complexes as well as for ZBr_3_ monomers additional calculations were performed where the aug-cc-pVTZ-PP basis set and an effective core potential (ECP) [62] were employed for the Br atom. The latter calculations were carried out to check if for the bromine systems analyzed the relativistic contribution to their properties is significant since this contribution is often detectable for heavier atoms. However, the comparison of the MP2/aug-cc-pVTZ results with those where relativistic corrections were introduced has shown only slight differences between them. The differences between distance parameters do not exceed 0.005 Å, between angles are lower than 0.2° while the differences between interaction and binding energies do not exceed 0.2 kJ/mol. This is why hereafter only MP2/aug-cc-pVTZ results without relativistic corrections for the bromine atom are discussed.

The abovementioned interaction energy and binding energy are defined in the following way. The interaction energy of the A–B complex is usually calculated according to the supermolecular approach [63]—Equation (1).

E_int_ = E_A…B_(A…B)^A^^∪B^ − E_A…B_(A)^A^ − E_A…B_(B) (1)

The designations in parentheses correspond to systems for which energies are calculated, the superscripts correspond to the basis sets used and the subscripts inform on the geometry optimized. Hence, the interaction energy is the difference between the energy of the A-B complex and the energies of A and B monomers. The geometry for the complex was optimized with the use the complex A∪B basis set. The monomers are characterized by geometries in the complex; energies for them were calculated with A and B monomers’ basis sets.

The interaction energy (Equation (1)) does not take into account the deformation energy being the result of the complexation. The definition of the binding energy where the deformation energy (E_def_) is taken into account has the following form.

E_bin_ = E_int_ + E_def_ = E_A…B_(A…B)^A^^∪B^ − E_A_(A)^A^ − E_B_(B)^B^ (2)

One can see that for the binding energy (Equation (2)), the energies of separately optimized monomers are considered (see the appropriate subscripts). The deformation energy defined by (Equation (3)) is positive since the separate molecules having the geometries taken from the complex are not in energetic minima.

E_def_ = E_A…B_(A)^A^ + E_A…B_(B)^B^ − E_A_(A)^A^ − E_B_(B)^B^ (3)

There are also the effects connected with the inconsistency of basis sets used for the whole complex and for the monomers. This is the well-known Basis Set Superposition Error (BSSE) which is positive and which decreases if the basis set applied is enlarged. The most often applied approach to assess BSSE is the counterpoise (CP) correction [64]. It is worth mentioning that recent reports indicate the corrected values could provide a larger error than the uncorrected ones [65,66]. Hence, the BSSE corrected and uncorrected interaction and binding energies for complexes analyzed are presented later here.

The Natural Bond Orbitals (NBO) method [34,67] implemented in the NBO 5.0 program [68] incorporated into GAMESS set of codes [69] was applied. The NBO method is used to characterize orbital-orbital interactions observed in the species analyzed here. The NBO atomic charges are also calculated and discussed in this study.

The Quantum Theory of “Atoms in Molecules” (QTAIM) [70] was applied for the localization of bond paths and corresponding critical points in complexes analyzed. The following characteristics of BCPs corresponding to the intermolecular interactions are considered here; the electron density at BCP (ρ_BCP_), its Laplacian (∇^2^ρ_BCP_) and the total electron energy density at BCP (H_BCP_). The QTAIM calculations were carried out with the use of AIMAll program [71].

## 4. Conclusions

The π-hole–π electrons interactions, *i.e.*, the triel bonds being the class of π-hole bonds, existing in complexes of the ZH_3_ and ZX_3_ triel species with acetylene and ethylene were analyzed. This was found that the BH_3_-C_2_H_2_ complex is characterized by the strong triel bond possessing numerous characteristics of the covalent bond. For the remaining complexes, the interactions between aluminum and π-electrons are much stronger than their boron counterparts. The former interactions may be also classified as partly covalent in nature since negative values of the total electron energy density at the bond critical point corresponding to the Al-π bond path are observed. The strength of the triel bonds in the BH_3_-C_2_H_2_ and aluminum complexes is connected with the large electron density shifts from the Lewis base (ethylene or acetylene) to the Lewis acid moiety; for the interactions in these complexes, the 3c-2e bond orbitals were found within the NBO approach. On the other hand, the negligible electron charge redistribution as a result of complexation is observed for the remaining boron complexes; note that the BF_3_-C_2_H_2_ and BF_3_-C_3_H_6_ complexes, analyzed before experimentally, were classified as van der Waals complexes [51] where the dispersive forces are dominant.

It was also found that the deformation energy increases with the strength of interaction, and that for the stronger bonded complexes, the geometrical changes tend to the tetravalent structures where the octet rule is obeyed. For weak interactions between the Z-center and π-electron system, the triel center may be considered a as trivalent one characterized by the hypovalency.

**Figure 7:**
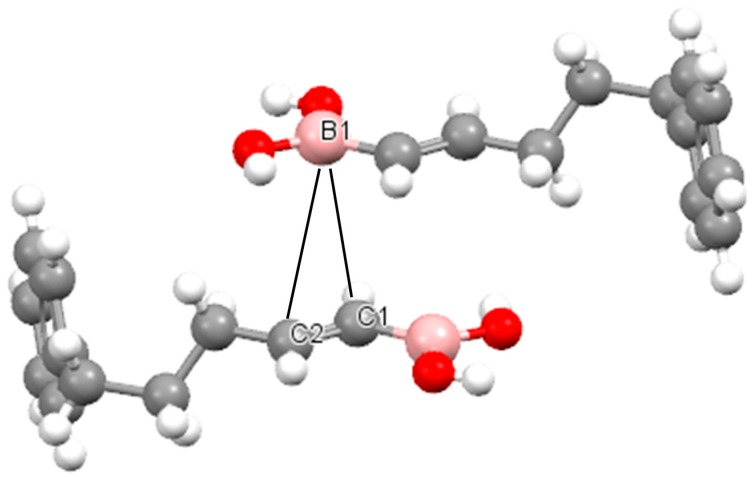
The fragment of the crystal structure of 5-phenylpent-1-en-1-yl)boronic acid, the boron-carbon contacts are shown (black solid lines) which correspond to the π-hole-π-electrons triel bond.

It is worth mentioning that the existence of π-hole-π electrons triel bonds described theoretically here may be confirmed experimentally. The Cambridge Structural Database (CSD) [72] search was performed and several structures with such interactions were found (the detailed study on the experimental evidences is in progress). Figure 7 shows an example of such a structure which was taken from CSD, the (5-phenylpent-1-en-1-yl)boronic acid structure [72] where the π-hole-π-electrons contacts are observed (B1…C1 and B1…C2 distances are equal to 3.47 and 3.70 Å).
